# Dual-Band All-Optical Logic Gates by Coherent Absorption in an Amorphous Silicon Graphene Metasurface

**DOI:** 10.3390/nano14040335

**Published:** 2024-02-08

**Authors:** Yixiao Chen, Chongyang Shen, Qingyuan Li, Jianyao Li, Xiaoxu Deng

**Affiliations:** School of Physics and Astronomy, Shanghai Jiao Tong University, Shanghai 200240, China; yixiaochen@sjtu.edu.cn (Y.C.); cy_shen@sjtu.edu.cn (C.S.); lqy1998@sjtu.edu.cn (Q.L.); ljy-sjtu@sjtu.edu.cn (J.L.)

**Keywords:** all-optical logic gates, metasurface, coherent absorption, multipole moments, graphene

## Abstract

The dual-band polarization-independent all-optical logic gate by coherent absorption effect in an amorphous silicon (a-Si) graphene metasurface is investigated theoretically and numerically. Taking the substrate effect into consideration, the coherent perfect absorption condition of the a-Si graphene metasurface is derived on the basis of the Cartesian multipole method. The coherent nearly perfect absorption of the a-Si graphene metasurface is realized by the interference of multipole moments and the interband transition of monolayer graphene, achieving peak values of 91% and 92% at 894.5 nm and 991.5 nm, respectively. The polarization independence of the coherent absorption is revealed due to the center symmetry of the structure of the a-Si graphene metasurface. The dual-band polarization-independent all-optical XOR and OR logic gates are implemented at 894.5 nm and 991.5 nm by the a-Si graphene metasurface based on the coherent nearly perfect absorption, which has the opportunity to be utilized in all-optical computing, all-optical data processing, and future all-optical networks.

## 1. Introduction

Optical logic gates, the fundamental element of optical computing, have attracted much attention due to their ultrahigh process speed, high information capacity, and low power consumption [1,2,3]. Metamaterials and their two-dimensional (2D) equivalents metasurfaces, are the artificial metal or dielectric structures that can manipulate the amplitude, phase, and polarization of electromagnetic waves in the subwavelength scale [4,5,6,7], which have been applied in a wide range of fields, including negative refraction [8], beam steering [9], holography [10], perfect absorption effect [5], sensing [6], etc. With the development of nanotechnology, metamaterials and metasurfaces have been used to realize optical logic gates for their advantages of integrability, broad operation bandwidth, and ability to manipulate light in the subwavelength scale [11,12,13]. Many methodologies of metamaterials and metasurfaces that are explored to achieve the optical logic gate have been investigated, such as the Fano resonance [13,14,15], diffractive neural network [1,16,17], coherent perfect absorption (CPA) [11,12,18], and so on. Significantly, coherent perfect absorption has aroused widespread attention because of the feature of all-optical phase-controllable modulation [19,20,21,22,23]. Using a free-standing planar metamaterial consisting of arrays of square split-ring aperture meta-molecules, the CPA-based all-optical multichannel logic gates and fiberized all-optical logic gates are demonstrated at 785 nm and 1550 nm by Zheludev’s group [11,12], respectively. By employing a graphene square ring as the unit cell of the metasurface, the CPA-based all-optical AND, OR, and XOR logic gates at 4.85 THz have been studied by Granpayeh’s group [18].

In this paper, the dual-band polarization-independent all-optical logic gate by coherent absorption effect in amorphous silicon (a-Si) graphene metasurface is theoretically and numerically studied. Based on the Cartesian multipole method, taking the substrate effect of coherent incident situations into account, the coherent perfect absorption condition of the a-Si graphene metasurface is derived. The effective multipole polarizabilities of the a-Si graphene metasurface are numerically calculated from COMSOL Multiphysics (COMSOL)-simulated displacement current density induced by multipole Mie resonances. The coherent absorption spectrum of a-Si graphene metasurface is numerically calculated by effective multipole polarizabilities and simulated by the Finite-Difference Time-Domain (FDTD) solutions, which have two absorption bands with peak values of 91% and 92% at 894.5 nm and 991.5 nm, respectively. By altering the Fermi energy of the monolayer graphene in the a-Si graphene metasurface, only the coherent absorption at 991.5 nm obviously changed, which is caused by the interband transition of the monolayer graphene. The coherent absorption is polarization-independent due to the central symmetry of the structure of the a-Si graphene metasurface. By setting the threshold intensity for the logic gate, the polarization-independent all-optical XOR and OR logic gates are implemented by the a-Si graphene metasurface at 894.5 nm and 991.5 nm based on the coherent nearly perfect absorption. The logic gate implemented by the a-Si graphene metasurface has the characteristic of dual-band operation, polarization independence, and ultrafast all-optical phase-controllability, which has the application prospect in all-optical computing, all-optical information processing, and next-generation optical networks.

## 2. Materials and Methods

**Figure 1:**
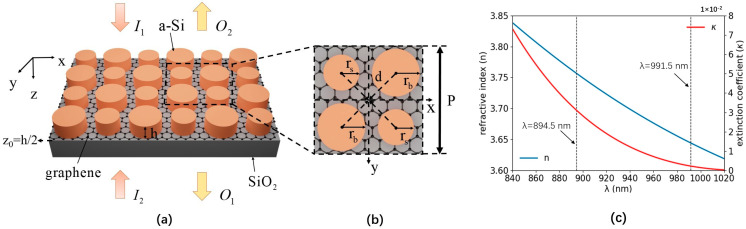

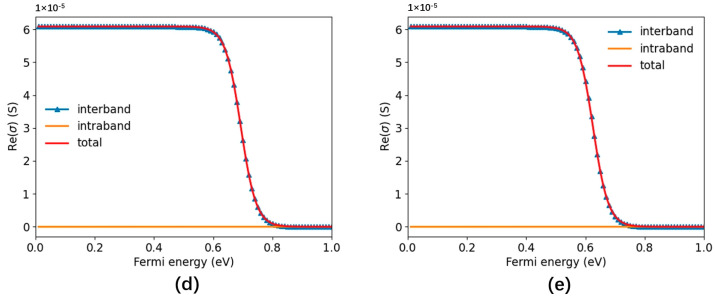
(**a**) Schematic of the dual-band polarization-independent coherent nearly perfect absorption amorphous silicon (a-Si) graphene metasurface. The black dashed parallelogram shows the unit cell. (**b**) Top view of the unit cell. (**c**) The complex refractive index n+iκ as a function of the wavelength of the amorphous silicon. (**d**,**e**) Real part of the complex conductivity σ as a function of the Fermi energy of the monolayer graphene at 894.5 and 991.5 nm, respectively.

The schematic of the dual-band polarization-independent coherent nearly perfect absorption a-Si graphene metasurface is shown in Figure 1a. The unit cell of the a-Si graphene metasurface, which comprises a tetramer cluster with two different sizes of a-Si nano-cylinders separated with the SiO_2_ substrate by a monolayer graphene, is shown in Figure 1b. Big and small nano-cylinders have radii of $r_{b}=176$ nm and $r_{s}=156$ nm, respectively. The period of the a-Si graphene metasurface is P=808 nm. The distance between the center of the nano-cylinder and the unit cell is d=202 nm. Amorphous silicon has a thickness of h=170 nm. The metasurface is placed in the vacuum. The complex refractive index of the amorphous silicon and SiO_2_ substrate is fitted by the data in Ref. [24]. The surface conductivity model of the monolayer graphene is described by Kubo formula [25]. In the simulation, periodic boundary condition was utilized along the x and y direction, and perfect match layer (PML) was set along the z direction. The auto non-uniform mesh with accuracy of 3 and physics-controlled mesh with extremely fine element size was used for the FDTD solutions and COMSOL, respectively. The plane wave source was used in all simulations.

The proposed fabrication process of the a-Si graphene metasurface will be started by depositing a monolayer graphene film on the SiO_2_ substrate by Plasma-Enhanced Chemical Vapor Deposition (PECVD). Secondly, an a-Si film can be grown on the graphene film by Low-Pressure Chemical Vapor Deposition (LPCVD) [26]. Thirdly, the photoresist can be spin-coated on the sample. Finally, Electron Beam Lithography (EBL) and Deep Silicon Etching (DSE) can be used to fabricate the metasurface.

Coherent light $E_{i1}$ and $E_{i2}=\alpha e^{i\left(\psi +kz\right)}E_{i1}$ are normally incident on the a-Si graphene metasurface in opposite directions, where α is the relative amplitude of $E_{i2}$ compared with $E_{i1}$, ψ is the initial phase difference between $E_{i1}$ and $E_{i2}$, and z is the phase reference point of two incident beams. The output fields on both sides of the a-Si graphene metasurface are $E_{o1}$ and $E_{o2}$, respectively, which can be obtained by incident fields from the scattering matrix:

(1) $$\left(\begin{array}{l} E_{o1}\\ E_{o2} \end{array}\right)=\left(\begin{array}{l} t_{1}\;r_{2}\\ r_{1}\;t_{2} \end{array}\right)\left(\begin{array}{l} E_{i1}\\ E_{i2} \end{array}\right)$$

where $t_{j}$ and $r_{j}$$\left(j=1,\;2\right)$ are the transmission and reflection coefficients of the +z$\left(j=1\right)$ and −z$\left(j=2\right)$ incident beams, respectively. The coherent absorption of the a-Si graphene metasurface is derived from Equation (1) [27]:

(2) $$A=1-\frac{{\left|r_{1}\right|}^{2}+\chi {\left|t_{1}\right|}^{2}+\alpha^{2}\left({\left|t_{2}\right|}^{2}+\chi {\left|r_{2}\right|}^{2}\right)+2\alpha \left[\left|r_{1}\right|\left|t_{2}\right|\cos(\psi +kz-\theta_{1})+\chi \left|t_{1}\right|\left|r_{2}\right|\cos(\psi +kz-\theta_{2})\right]}{1+\chi \alpha^{2}}$$

where $\theta_{1}$ is the phase difference between $r_{1}$ and $t_{2}$; $\theta_{2}$ is the phase difference between $t_{1}$ and $r_{2}$; $\chi =\sqrt{\varepsilon_{2}/\varepsilon_{1}}$ (where $\varepsilon_{1}$ and $\varepsilon_{2}$ are the relative permittivity of the surrounding medium and the substrate, respectively). When ψ+kz=M+2Nπ (where *M* is a constant) and $\psi +kz=M+\left(2N+1\right)\pi$, the coherent absorption A achieves maximum $A_{co,\max}$ and minimum $A_{co,\min}$ at the condition of α, $t_{j}$, and $r_{j}$ are fixed. By adjusting Δφ≡ψ+kz (where ψ can be dynamically tuned by an external phase shifter), the value of coherent absorption A of the a-Si graphene metasurface can be adjusted flexibly from $A_{co,\min}$ to $A_{co,\max}$.

The coherent absorption effect of the a-Si graphene metasurface is further investigated based on the Cartesian multipole method. Coherent laser-induced displacement current density is generated in the a-Si graphene metasurface. According to the Cartesian multipole method, the electric dipole moment (ED), the magnetic dipole moment (MD), the electric quadrupole moment (EQ), the magnetic quadrupole moment (MQ), and the electric octupole moment (EO) of the a-Si graphene metasurface’s unit cell are calculated by the displacement current density [28], and other moments are ignored. Under the x-polarization of the incident beams, the multipole moments of the a-Si graphene metasurface’s unit cell are only contributed by the $p_{x}$, $m_{y}$, $Q_{xz}$, $M_{yz}$, and $O_{xzz}$ components [29].

Considering the different substrate effects on opposite incident beams, the transmission ($t_{1}$, $t_{2}$) and reflection ($r_{1}$, $r_{2}$) coefficients of the a-Si graphene metasurface are contributed by the far-field scattering lights, which are induced by the multipole moments [29,30,31,32,33]:

(3) $$\begin{array}{l} t_{1}=\left[1+\frac{ik_{1}}{2S_{L}}\left(\alpha_{eff,1}^{p}+\alpha_{eff,1}^{m}-\alpha_{eff,1}^{Q}-\alpha_{eff,1}^{M}-\alpha_{eff,1}^{O}\right)\right]A\\ r_{1}=\frac{ik_{1}}{2S_{L}}\left[\left(1+B\right)\left(\alpha_{eff,1}^{p}-\alpha_{eff,1}^{M}-\alpha_{eff,1}^{O}\right)-\left(1-B\right)\left(\alpha_{eff,1}^{m}-\alpha_{eff,1}^{Q}\right)\right]+B\\ t_{2}=\frac{ik_{1}}{2S_{L}}\left[\left(1+B\right)\left(\alpha_{eff,2}^{p}-\alpha_{eff,2}^{M}-\alpha_{eff,2}^{O}\right)-\left(1-B\right)\left(\alpha_{eff,2}^{m}-\alpha_{eff,2}^{Q}\right)\right]+C\\ r_{2}=\frac{ik_{1}}{2S_{L}}\left(\alpha_{eff,2}^{p}+\alpha_{eff,2}^{m}-\alpha_{eff,2}^{Q}-\alpha_{eff,2}^{M}-\alpha_{eff,2}^{O}\right)A+D \end{array}$$

where $\alpha_{eff,j}^{p}=p_{x,j}/E_{0}\varepsilon_{0}\varepsilon_{1}$, $\alpha_{eff,j}^{m}=m_{y,j}/v_{1}E_{0}\varepsilon_{0}\varepsilon_{1}$, $\alpha_{eff,j}^{Q}=ik_{1}Q_{xz,j}/6E_{0}\varepsilon_{0}\varepsilon_{1}$, $\alpha_{eff,j}^{M}=ik_{1}M_{yz,j}/2v_{1}E_{0}\varepsilon_{0}\varepsilon_{1}$, and $\alpha_{eff,j}^{O}=k_{1}^{2}O_{xzz,j}/6E_{0}\varepsilon_{0}\varepsilon_{1}$ are the effective ED, MD, EQ, MQ, and EO polarizability, respectively; $A=t_{0,1}e^{i\left(k_{1}-k_{2}\right)z_{0}}$; $B=r_{0,1}e^{2ik_{1}z_{0}}$; $C=t_{0,2}e^{i(k_{1}-k_{2})z_{0}}$; and $D=r_{0,2}e^{-2ik_{2}z_{0}}$. $t_{0,j}$ and $r_{0,j}$ are the Fresnel transmission and reflection coefficient, respectively. $k_{1}=k_{0}\sqrt{\varepsilon_{1}}$ and $k_{2}=k_{0}\sqrt{\varepsilon_{2}}$ ($k_{0}$ is the wave number in the vacuum) are the wave number in the surrounding medium and the substrate, respectively. $\varepsilon_{0}$ is the vacuum permittivity; $v_{1}$ is the light speed in the surrounding medium; $z_{0}=h/2$ is the position of the graphene–substrate interface; $S_{L}$ is the area of the unit cell; $E_{0}$ is the electric field of the incident wave at the point of the multipole expansion center.

Setting Δφ=0 and α=1, taking Equation (3) into Equation (2), when the effective multipole polarizabilities satisfy:

(4) $$\begin{array}{l} \alpha_{eff,t}^{p}-\alpha_{eff,t}^{M}-\alpha_{eff,t}^{O}-O_{1}=0\\ \alpha_{eff,t}^{m}-\alpha_{eff,t}^{Q}-O_{2}=0\\ O_{1}\equiv \frac{S_{L}}{ik_{1}}\left(\frac{BD-AC}{A}-1-\frac{D}{A}\right)\\ O_{2}\equiv \frac{S_{L}}{ik_{1}}\left(\frac{AC-BD}{A}-1-\frac{D}{A}\right) \end{array}$$

the coherent perfect absorption is achieved, where $\alpha_{eff,t}^{multi}=\alpha_{eff,1}^{multi}+\alpha_{eff,2}^{multi}$ (*multi = p*, *m*, *Q*, *M*, and *O*). The coherent outputs for both sides are suppressed due to the destructive interference between the multipole resonances, achieving coherent nearly perfect absorption of the a-Si graphene metasurface.

**Figure 2:**
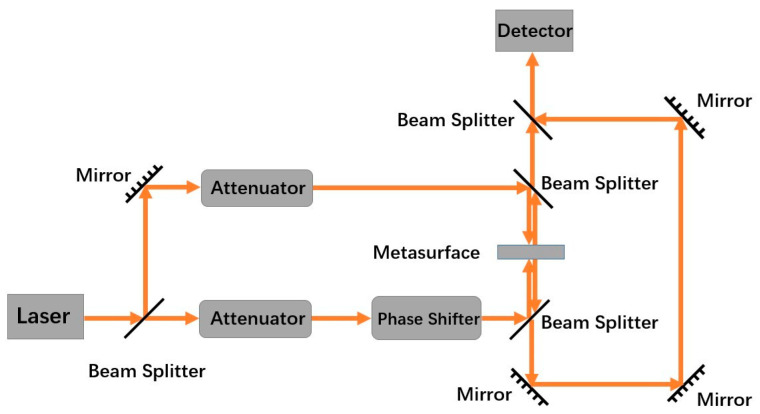
The optical path diagram for the metasurface-based all-optical logic gate.

The coherent absorption effect of the a-Si graphene metasurface is employed to realize the all-optical logic gate. The optical path diagram for the metasurface-based all-optical logic gate is shown in Figure 2, which is similar to the optical path in Ref. [20]. The intensity of the incident beam $I_{in}$ (where the intensities of two incident beams are set to equal) and the total output intensity $I_{out}$ of the a-Si graphene metasurface are considered as the input state and output state of the logic gate, respectively. Setting the intensity threshold

(5) $$I_{th}=\left[\left(1-A_{single,min}\right)+2\left(1-A_{co,\max,△\varphi =0}\right)\right]I_{in}/2$$

that is, if $I_{in}$ or $I_{out}$ is higher than $I_{th}$, the logic is 1, and if it is lower than $I_{th}$, the logic is 0. When the phase difference between two coherent beams △φ=0 (that is, the coherent nearly perfect absorption is achieved), the output logic of the a-Si graphene metasurface is 0 whether the input state is coherent input (1, 1) or zero input (0, 0), while the output logic is 1 whether the input state is +z input (1, 0) or −z input (0, 1), realizing the all-optical XOR logic gate. When △φ=π (that is, the coherent absorption is almost non-existent), the output logic of the a-Si graphene metasurface is 0 when the input state is (0, 0), while the output logic is 1 whether the input state is (1, 1), (1, 0), or (0, 1), realizing the all-optical OR logic gate. The proposed metasurface achieves all-optical logic at two bands under the same intensity threshold, where both of these bands can realize coherent nearly perfect absorption.

## 3. Results and Discussion

### 3.1. Coherent Nearly Perfect Absorption in the a-Si Graphene Metasurface

**Figure 3:**
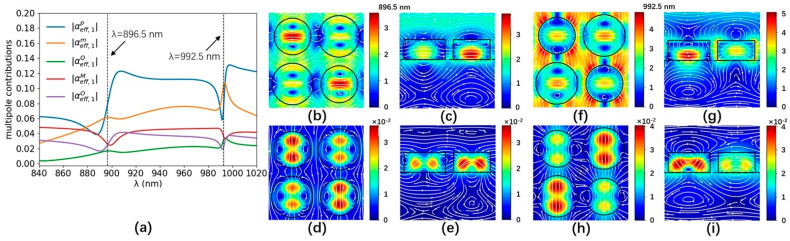
Absolute values of the multipole contributions of the a-Si graphene metasurface for +z incident beam (**a**). The blue, orange, green, red, and purple lines are the ED, MD, EQ, MQ, and EO contribution, respectively. The images show the electric fields and their vector distributions of the a-Si graphene metasurface in the x-y plane (z = 0 nm) at 896.5 nm (**b**) and 992.5 nm (**f**), in the x-z plane (y = 200 nm) at 896.5 nm (**c**) and 992.5 nm (**g**) for the +z incident beam. The images show the magnetic fields and their vector distributions of the a-Si graphene metasurface in the x-y plane (z = 0 nm) at 896.5 nm (**d**) and 992.5 nm (**h**), in the y-z plane (x = 200 nm) at 896.5 nm (**e**) and 992.5 nm (**i**) for the +z incident beam. The circles and boxes represent the boundary of the a-Si nano-cylinders.

**Figure 4:**
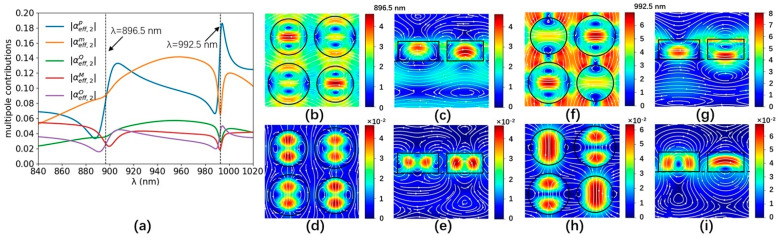
Absolute values of the multipole contributions of the a-Si graphene metasurface for −z incident beam (**a**). The blue, orange, green, red, and purple lines are the ED, MD, EQ, MQ, and EO contribution, respectively. The images show the electric fields and their vector distributions of the a-Si graphene metasurface in the x-y plane (z = 0 nm) at 896.5 nm (**b**) and 992.5 nm (**f**), in the x-z plane (y = 200 nm) at 896.5 nm (**c**) and 992.5 nm (**g**) for the −z incident beam. The images show the magnetic fields and their vector distributions of the a-Si graphene metasurface in the x-y plane (z = 0 nm) at 896.5 nm (**d**) and 992.5 nm (**h**), in the y-z plane (x = 200 nm) at 896.5 nm (**e**) and 992.5 nm (**i**) for the −z incident beam. The circles and boxes represent the boundary of the a-Si nano-cylinders.

**Figure 5:**
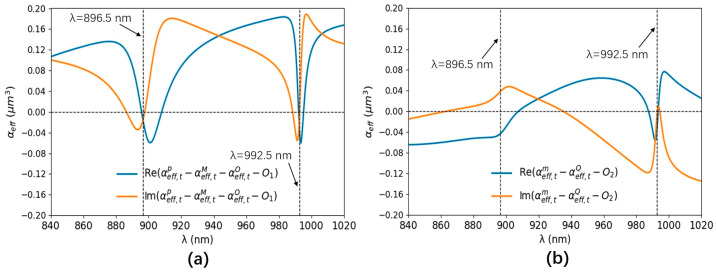
The real part (blue line) and image part (orange line) of the effective polarizabilities $\alpha_{eff,t}^{p}-\alpha_{eff,t}^{M}-\alpha_{eff,t}^{O}-O_{1}$ (**a**) and $\alpha_{eff,t}^{m}-\alpha_{eff,t}^{Q}-O_{2}$ (**b**).

The multipole contributions of the a-Si graphene metasurface are numerically analyzed by the Cartesian multipole method. The laser-induced displacement current density of the a-Si graphene metasurface is simulated by Finite Element Method (COMSOL). The simulated parameters are the same as Figure 1. The scattering rate and simulation temperature of the graphene in the Kubo formula are set to 0.00017 eV and 300 K, respectively. The +z and −z incident laser beams are normally incident on the a-Si graphene metasurface with x-polarization, respectively. The effective multipole polarizabilities in Equation (3) are numerically calculated by the equation of multipole moments in Ref. [28] in the spectral range from 840 nm to 1020 nm, which is shown in Figure 3a and Figure 4a, respectively. The multipole resonant wavelength occurs at ~896.5 nm and ~992.5 nm. The maps of the resonant electromagnetic fields and their vector distribution of the a-Si graphene metasurface at 896.5 nm and 992.5 nm are simulated by FDTD solutions, which are plotted in Figure 3b–i and Figure 4b–i, respectively. The real part and image part of the effective polarizabilities $\alpha_{eff,t}^{p}-\alpha_{eff,t}^{M}-\alpha_{eff,t}^{O}-O_{1}$ and $\alpha_{eff,t}^{m}-\alpha_{eff,t}^{Q}-O_{2}$ in Equation (4) are calculated and shown in Figure 5a,b, respectively. The values of $\alpha_{eff,t}^{p}-\alpha_{eff,t}^{M}-\alpha_{eff,t}^{O}-O_{1}$ and $\alpha_{eff,t}^{m}-\alpha_{eff,t}^{Q}-O_{2}$ at two resonant wavelengths basically satisfy Equation (4), the small discrepancy between them and zero is due to the multipole moments generated by the a-Si graphene metasurface are not completely overlapping, therefore, the coherent nearly perfect absorption peaks are achieved at ~896.5 nm and ~992.5 nm.

**Figure 6:**
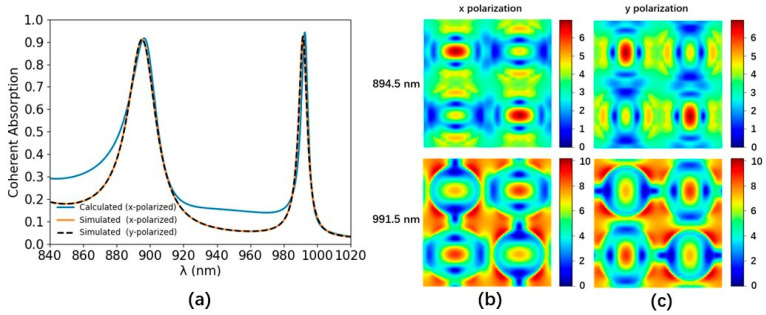
(**a**) The calculated absorption spectra of the a-Si graphene metasurface under coherent illumination with x-polarization (blue line). The simulated absorption spectra of the a-Si graphene metasurface under coherent illumination with x-polarization (orange line) and y-polarization (black dashed line). (**b**) The electric field at resonance wavelengths under coherent illumination with x-polarization in the x-y plane (z = 0 nm). (**c**) The electric field at resonance wavelengths under coherent illumination with y-polarization in the x-y plane (z = 0 nm).

The coherent absorption of the a-Si graphene metasurface is numerically calculated based on effective multipole polarizabilities by Equation (3) shown as the blue line in Figure 6a, while that simulated by FDTD solutions is the orange one. The phase difference between two coherent beams △φ and the relative amplitude α are set to 0 and 1, respectively. The calculated coherent absorption peaks appear at 896.5 nm and 992.5 nm with an absorption of 92% and 94%, respectively, which corresponds to the resonance points of multipole moments. The simulated maximum coherent absorption is 91% and 92% at 894.5 nm and 991.5 nm, respectively. The small discrepancy between the simulation and the calculation might arise from the incompleteness of the multipole expansion in the Cartesian multipole method we have used [31]. When the temperature increases, the extinction coefficient κ of amorphous silicon will increase, suppressing the scattered light of the a-Si graphene metasurface [34], resulting in an increase in absorption.

**Figure 7:**
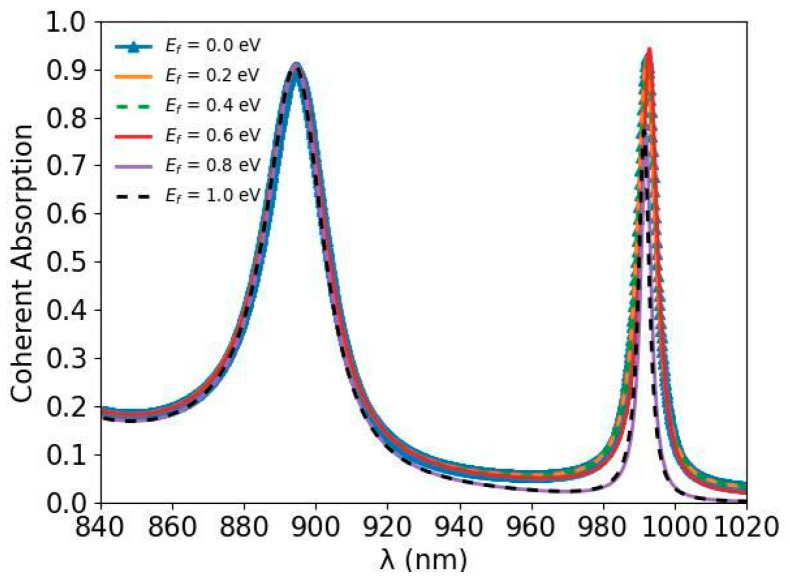
The simulated coherent absorption spectra of the a-Si graphene metasurface with different Fermi energies.

The coherent absorption spectra of the a-Si graphene metasurface with various Fermi energies of the monolayer graphene are simulated by the FDTD solutions and shown in Figure 7. With the increasing of the Fermi energy ($E_{f}$), the conductivity of interband transition [35,36] in Kubo formula has a plunge around $E_{f}=hc/2\lambda$, which influences the coherent absorption of the a-Si graphene metasurface. Both absorption peaks at 894.5 nm and 991.5 nm slightly red shift when $E_{f}<hc/2\lambda$, and blue shift $E_{f}>hc/2\lambda$. At the 991.5 nm resonant point, the coherent absorption reduces from ~92% to ~77% when $E_{f}$ changes from $E_{f}<hc/2\lambda$ to $E_{f}>hc/2\lambda$, while the coherent absorptions at 894.5 nm are almost not changed. The real part of the complex conductivity σ of the monolayer graphene has a slight difference at two resonance wavelengths due to the dispersion which is shown in Figure 1d,e, and the extinction coefficient κ of amorphous silicon at 894.5 nm is almost 14 times that at 991.5 nm which is shown in Figure 1c, leading to the different electric field distribution at the monolayer graphene which affects the coherent absorption of the a-Si graphene metasurface.

The coherent absorption spectrum of y-polarization incident coherent beams is also simulated by FDTD solutions and is shown as the black dashed line in Figure 6a. The maps of the simulated electric field distribution of the a-Si graphene metasurface in the x-y plane (z = 0 nm) at the resonance wavelength of 894.5 nm and 991.5 nm under x and y polarization incident beams are shown in Figure 6b,c. Although the simulated electrical field distributions rotate 90 degrees with the polarization of incident beams change from x to y, the coherent absorption is almost unchanged due to the central symmetry of the a-Si graphene metasurface. Therefore, the coherent absorption of the a-Si graphene metasurface is polarization-independent.

**Figure 8:**
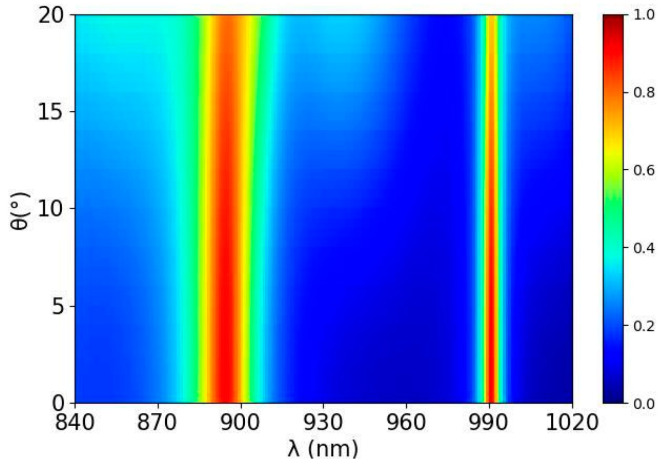
Simulated coherent absorption spectra of the a-Si graphene metasurface with different incident angle θ (θ represents incident angle, and θ =0° represents normal incidence).

The coherent absorption of the a-Si graphene metasurface is simulated with different incident angle in spectral range from 840 nm to 1020 nm by FDTD solutions and shown in Figure 8. When the incident angle θ increases form 0° to 10°, both absorption peaks at 894.5 nm and 991.5 nm present slight redshift, and the peak values have decreased but are less than 7%, maintaining the property of coherent nearly perfect absorption.

### 3.2. Dual-Band All-Optical Metasurface-Based Logic Gates

The dual-band XOR and OR all-optical logic gates have been analyzed based on the coherent nearly perfect absorption effect of the a-Si graphene metasurface. The input intensity and the FDTD-simulated output intensity of the a-Si graphene metasurface at 894.5 nm and 991.5 nm are shown in Table 1. Based on Equation (5), the intensity threshold of a-Si graphene metasurface is calculated as $I_{th}=0.27I_{in}$, and the input and output logic in different situations are confirmed and shown in Table 2. According to Table 2, the dual-band XOR and OR logic is established when the phase difference between the coherent beams Δφ= 0 (which corresponds to coherent nearly perfect absorption) and Δφ=π (which corresponds to coherent absorption is almost non-existent), respectively. The all-optical phase-controlled logic gate is realized by the proposed metasurface on the basis of coherent absorption.

The performance of the proposed logic gate in comparison with the existing metasurface-based logic gates is presented in Table 3. The proposed metasurface-based logic gate has the properties of dual-band operation and polarization independence, which are induced by the dual-band coherent nearly perfect absorption and structural symmetry of the a-Si graphene metasurface.

## 4. Conclusions

In summary, the near-infrared dual-band polarization-independent all-optical logic gate is investigated theoretically and numerically by the a-Si graphene metasurface based on coherent absorption. Considering the substrate effect of opposite incident situations, the coherent perfect absorption condition of the a-Si graphene metasurface is analyzed based on the Cartesian multipole method. The simulated coherent absorption spectrum of the a-Si graphene metasurface realizes the peak values of 91% and 92% at 894.5 nm and 991.5 nm, respectively. The influence of the monolayer graphene on the coherent absorption of the a-Si graphene metasurface is studied. The center symmetry of the a-Si graphene metasurface’s structure causes the polarization independence of the coherent absorption. By introducing the threshold intensity for the logic gate, the all-optical polarization-independent XOR and OR logic gates are studied based on coherent nearly perfect absorption at 894.5 nm and 991.5 nm by the a-Si graphene metasurface. The proposed dual-band polarization-independent all-optical logic gate realized by the a-Si graphene metasurface has promising prospects in all-optical computing, all-optical data processing, and future optical information networks.

## Figures and Tables

**Table 1:** The input and output intensity of the a-Si graphene metasurface in different situations.

Input State	Input Intensity		Output Intensity at 894.5 nm		Output Intensity at 991.5 nm	
			Δφ=0	Δφ=π	Δφ=0	Δφ=π
coherent incident	$I_{in}$	$I_{in}$	0.18$I_{in}$	1.36$I_{in}$	0.16$I_{in}$	1.70$I_{in}$
+z incident	$I_{in}$	0	0.44$I_{in}$	0.44$I_{in}$	0.62$I_{in}$	0.62$I_{in}$
−z incident	0	$I_{in}$	0.36$I_{in}$	0.36$I_{in}$	0.36$I_{in}$	0.36$I_{in}$
zero incident	0	0	0	0	0	0

**Table 2:** The input and output logic for the metasurface-based logic gate in different situations.

Input State	Input Logic		Output Logic at 894.5 nm		Output Logic at 991.5 nm	
			Δφ=0	Δφ=π	Δφ=0	Δφ=π
coherent incident	1	1	0	1	0	1
+z incident	1	0	1	1	1	1
−z incident	0	1	1	1	1	1
zero incident	0	0	0	0	0	0

**Table 3:** Comparison between our work and previous works.

Methodologies	Logic Functions	Operation Wavelength or Frequency	Polarization Independence	Modulation Type
Fano resonant [15]	NOT, XOR, XNOR, NAND, OR	0.53 THz, 0.56 THz	no	Voltage modulation
Diffractive neural network [1]	NOT, OR, AND	17 GHz	no	Spatially encode
Coherent perfect absorption [11]	XOR, XNOR, AND, OR	785 nm	no	Phase control, Spatially encode
Coherent perfect absorption [18]	XOR, AND, OR	4.85 THz	yes	Phase control
Our works	XOR, OR	894.5 nm, 991.5 nm	yes	Phase control

## Data Availability

Data are contained within the article.
